# Determining chromosomal arms 1p/19q co-deletion status in low graded glioma by cross correlation-periodogram pattern analysis

**DOI:** 10.1038/s41598-021-03078-1

**Published:** 2021-12-13

**Authors:** Debanjali Bhattacharya, Neelam Sinha, Jitender Saini

**Affiliations:** 1 Networking and Communication, International Institute of Information Technology, Bangalore, 560100 India; 2 Neuroimaging and interventional radiology, National Institute of Mental Health and Neuro Science, Bengaluru, 560029 India

**Keywords:** Cancer, Neuroscience, Biomarkers, Diseases, Health care, Medical research, Neurology, Oncology, Engineering

## Abstract

Prediction of mutational status of different graded glioma is extremely crucial for its diagnosis and treatment planning. Currently FISH and the surgical biopsy techniques are the ‘gold standard’ in the field of diagnostics; the analyses of which helps to decide appropriate treatment regime. In this study we proposed a novel approach to analyze structural MRI image signature pattern for predicting 1p/19q co-deletion status non-invasively. A total of 159 patients with grade-II and grade-III glioma were included in the analysis. These patients earlier underwent biopsy; the report of which confirmed 57 cases with no 1p/19q co-deletion and 102 cases with 1p/19q co-deletion. Tumor tissue heterogeneity was investigated by variance of cross correlation (VoCC). Significant differences in the pattern of VoCC between two classes was quantified using Lomb-Scargle (LS) periodogram. Energy and the cut-off frequency of LS power spectral density were derived and utilized as the features for classification. RUSBoost classifier was used that yield highest classification accuracy of 84% for G-II and 87% for G-III glioma respectively in classifying 1p/19q co-deleted and 1p/19q non-deleted glioma. In clinical practice the proposed technique can be utilized as a non-invasive pre-confirmatory test of glioma mutation, before wet-lab validation.

## Introduction

Low graded glioma (LGG) are the group of primary brain tumor that are produced from two different types of glial cells of brain called astrocytes and oligodendrocytes and are termed as astrocytomas and oligodendrogliomas respectively^1^. Compared to high-grade glioma (anaplastic astrocytoma, and oligodendroglioma, glioblastoma), LGG being less aggressive, if detected early can lead to better survival rates. However there are several factors that influence the glioma progression. In last few years deeper genetic analyses on large number of glioma samples have led to the discovery of the ’genetic risk factor’, which plays a key role in glioma prognosis^1^. Hence determining the mutational status is rapidly becoming an integral part of the routine pathological study of gliomas that provide both diagnostic and prognostic information. At present, the popular approaches for determining mutational status are DNA sequencing, immune-histochemical staining and FISH test^2^. But all of these techniques are invasive in nature. Thus the objective of our study is to explore a novel approach that would be able to predict mutational status across diverse glioma grades in a non-invasive manner. In this regard we have determined chromosomal arms 1p/19q co-deletion status in grade-II (G-II) and grade-III (G-III) glioma which is one of the most common cancer drivers in glioma and widely used as a strong prognostic biomarker in gene mutation study of glioma. The 1p/19q co-deletion stands for the combined loss of the short arm chromosome 1 (i.e. 1p) and the long arm of chromosome 19 (i.e. 19q). Co-deletion of 1p/19q is observed to be associated with relatively improved survival rate in comparison to tumors with non-deletion, irrespective of tumor morphology or histologic grade. Several studies on glioma mutation have found that the 1p/19q co-deletion in LGG gives positive response towards treatment and is associated with progression-free survival. Hence the prediction of 1p/19q status in LGG patients is extremely crucial for development of effective treatment strategies. Numerous imaging assays have been executed to determine the molecular characteristics and prognostic markers in LGG using multi-modal medical images^3–6^. But many of these studies were not able to show satisfactory results in order to determine the 1p/19q co-deletion status in individual patients.

In the current study we have performed non-invasive determination of 1p/19q mutational status by assessing the volumetric tumor heterogeneity in mutated (1p/19q co-deleted) and wildtype (1p/19q non-deleted) gliomas from multi-contrast structural MRI (S-MRI) images. One of our previous works on glioma mutation^7^ showed that the inter-slice texture pattern of 86% of grade-III (G-III) gliomas and 90% of grade-II (G-II) gliomas that occurred due to mutation are homogeneous in nature; while it was found to be random and heterogeneous for most of the wildtype cases (70% of total cases). Thus the aim of the present study is to model the textural characteristics of glioma tissue using S-MRI images that would be able to differentiate between mutated and wildtype gliomas. To achieve this, we have proposed a new *“Cross correlation-Periodogram Model”* to determine the 1p/19q co-deletion status non-invasively. In clinical practice the proposed technique can be potentially used as a non-invasive pre-confirmatory test of glioma mutation which could serve as an alternative to surgical biopsy and histopathological analysis.

**Figure 1:**
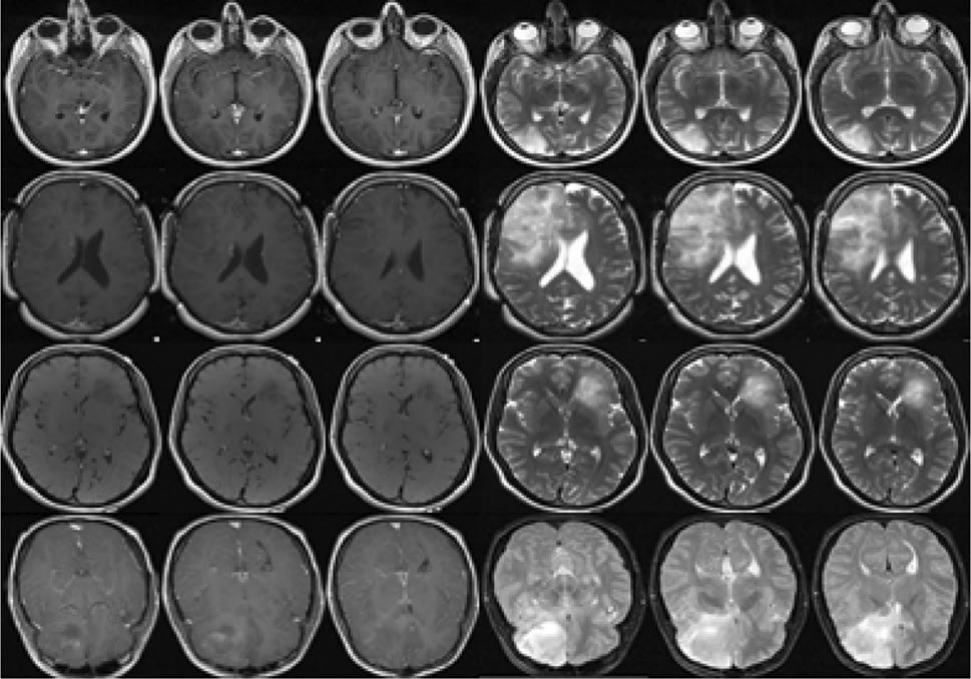
Representative MRI images for four subjects are shown. Column-1 to column-3 are post contrast T1-W MRI; column-4 to column-6 are T2-W MRI. Row-1 and row-2 represent 1p/19q co-deleted G-II and G-III glioma respectively. Row-3 and row-4 represent 1p/19q non-deleted G-II and G-III glioma respectively.

**Figure 2:**
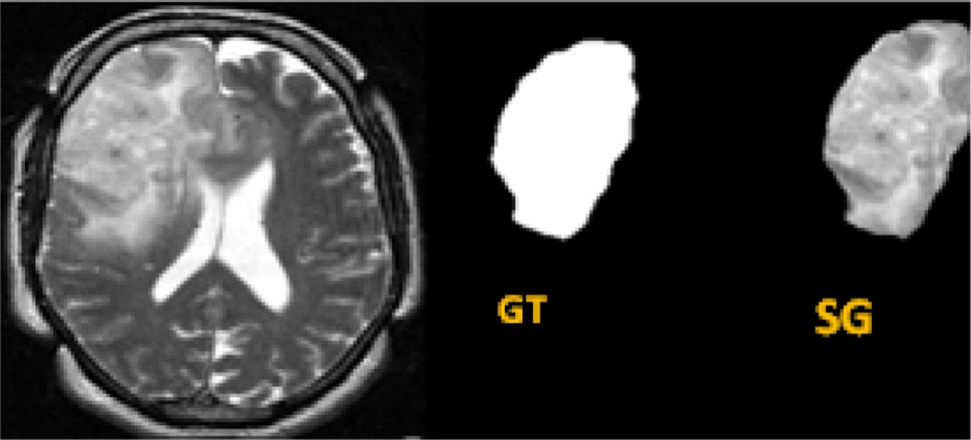
Glioma extraction from MRI using the ground truth. Here the abbreviations GT and SG represent the ground truth and segmented glioma respectively.

**Figure 3:**
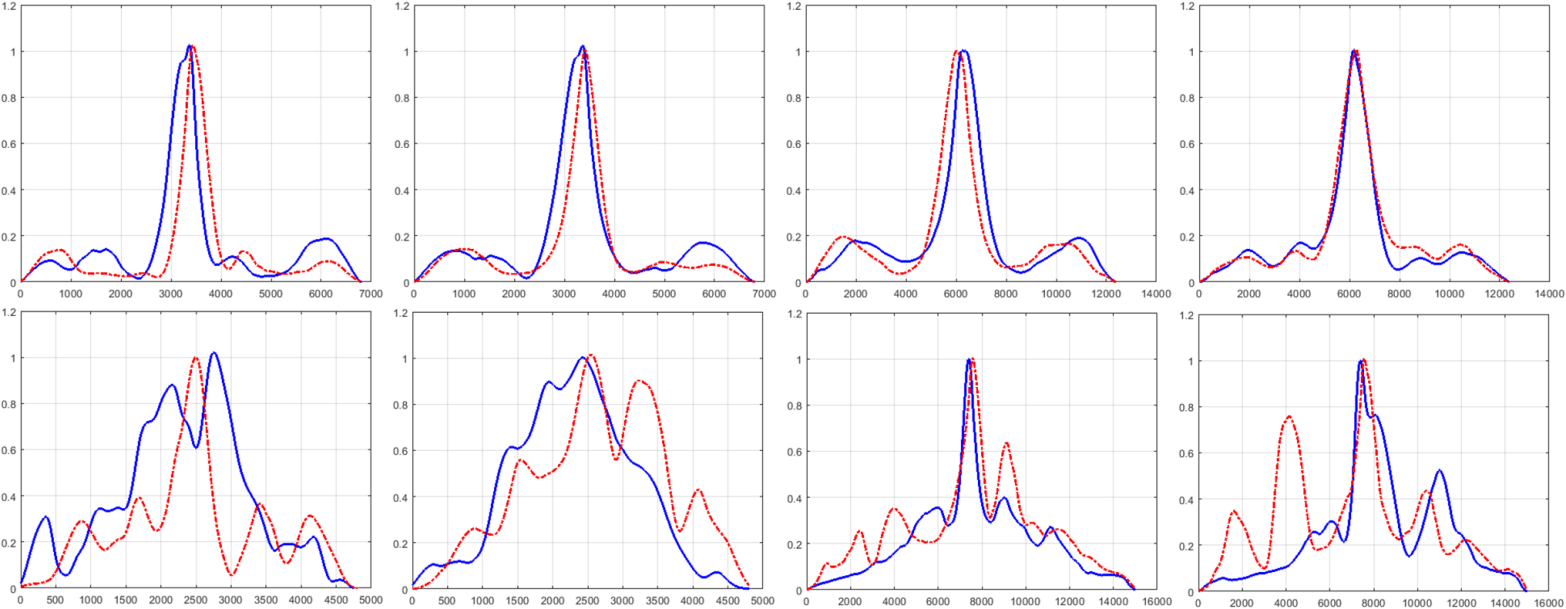
Changes in variance of cross correlation (VoCC) curves across consecutive MRI slices (VoCC between slice-1 and slice-2 was shown by thick ’Blue’ curve, whereas VoCC between slice-2 and slice-3 was shown by dotted ’Red’ curve) of LGG are plotted with 1p/19q co-deletion (Top row) and 1p/19q non-deletion (Bottom row). Column 1 and column 2 plots the same for G-II glioma with T1-W and T2-W MRI respectively and column 3 and column 4 plots the same for G-III glioma with T1-W and T2-W MRI respectively. As seen from the graph, the pattern of VoCC changes drastically across slices for wildtype cases whereas the across-slice VoCC pattern is similar for glioma that occurred due to mutation.

**Figure 4:**
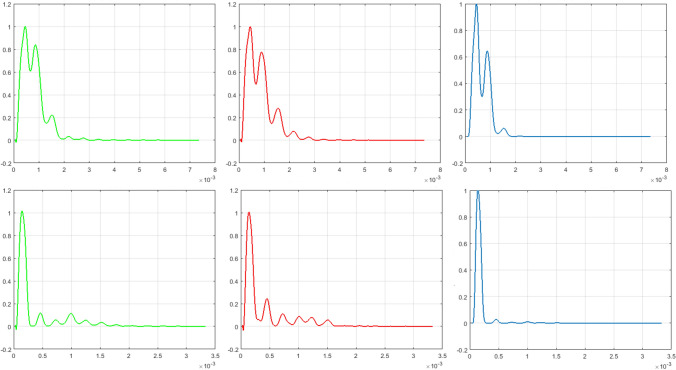
LSPSD estimation of VoCC for one representative glioma subject having 1p/19q co-deletion (top row) and one representative glioma subject having 1p/19q non-deletion (bottom row) are showed. The ’Green’ curve shows the LS periodogram of VoCC between slice-1 and slice-2. The ’Red’ curve shows the LS periodogram of VoCC between slice-2 and slice-3. The inner product of two periodogram (shown in ’Blue’) reveals the difference in volumetric periodicity of two classes.

**Figure 5:**
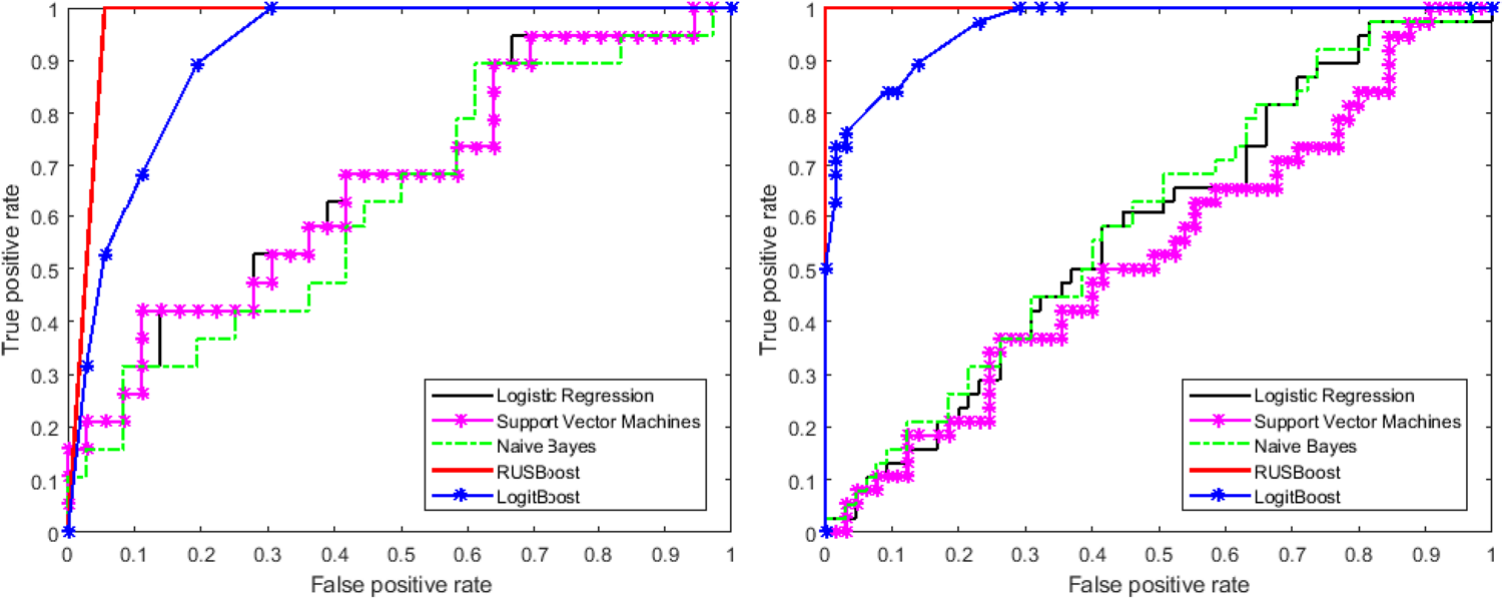
Illustration of receiver operating characteristics (ROC) curve for different classifiers. The best training accuracy was obtained using RUSBoost with AUC of 0.98 (G-II) and 1 (G-III).

## Results

### Dataset description

Structural MRI images of glioma are acquired from “The Cancer Imaging Archive (TCIA)” online database^8^, available for public access. It contains 2 different contrasts, which are 1mm thick sliced T1 post contrast (T1C) and 3mm thick sliced T2-weighted images of G-II and G-III gliomas. A total of one hundred fifty nine (*n=159,* diagnosed between 01 October 2002 to 01 August 2011) LGG patients, diagnosed with pre-operative G-II and G-III glioma and having biopsy proven 1p/19q co-deletion and non-deletion were used in this study. The dataset consists of MRI brain images of 57 non-deleted (*n=38* for G-II; *n=19* for G-III) and 102 co-deleted (*n=65* for G-II; *n=36* for G-III) glioma subjects. The dataset also contains the ground truth of segmented glioma of 3 consecutive slices in each subject including the one with the biggest tumor diameter and ones just below and above it. Hence a total of 477 slices (3 slices per LGG subject) were incorporated in this study. All images were acquired at 1.5T or 3T on either Siemens Medical System (Malvern, PA, USA) or General Electric Medical System (Waukesha, WI, USA) scanner. Representative T2-W and T1C-W glioma MRI images having 1p/19q co-deletion and 1p/19q non-deletion are shown in Fig. 1. Glioma segmentation was performed using the ground truth provided by TCIA. The segmented glioma from whole MRI is shown in Fig. 2 along with the ground truth image. The across-slice behaviour of 1p/19q co-deleted and 1p/19q non-deleted glioma is discussed in the subsequent subsections.

The entire code was executed in MATLAB, version R2018a, and run on a machine with Intel-R Core I3 4005U CPU 1.70 GHz processor with 4.0 GB RAM.

### Consistency in variance of cross correlation pattern across slices for 1p/19q co-deleted glioma cases

The change in glioma heterogeneity across MRI slices are captured by computing variance of cross correlation (VoCC) of two consecutive slices. The VoCC of 3 successive glioma slices at different lags were plotted for the two classes having (i) 1p/19q co-deletion and (ii) 1p/19q non-deletion and this is shown in Fig. 3. As seen from Fig. 3 in case of 1p/19q co-deleted subjects the derived VoCC metric showed consistency in the VoCC pattern between the two consecutive slices, while for the wild type cases a random pattern in VoCC was observed across slices. High variation in VoCC pattern for wildtype glioma cases indicate the change in across-slice heterogeneity, whereas the similarity with relatively low variation in VoCC for mutated glioma cases indicates homogeneity across glioma slices.

### Lomb-Scargle power spectral density reveals static periodicity across volumetric glioma slices having 1p/19q co-deletion

The noted difference in VoCC (Fig. 3) was analyzed using the periodogram spectral analysis where the energy and the cut-off frequency of Lomb-Scargle power spectral density (LSPSD) estimate were used as features for classification. The LSPSD estimate of VoCC for one glioma subject with 1p/19q co-deletion and non-deletion is shown in Fig. 4. For 1p/19q co-deleted glioma cases the observed similarity in LSPSD across consecutive slices reveals the presence of static periodicity. However for 1p/19q non-deleted cases we did not find any static periodic pattern across slices. Thus, unlike 1p/19q co-deleted glioma subjects in case of 1p/19q non-deleted subjects the inner product of two LSPSD estimate could not show any dominant periodic component in the glioma volume. This characteristic, seen in wildtype cases, in turn lowers the cut-off frequency values and maximize the difference in energy across consecutive MR slices. It has to be noted that the first peak of periodogram that was observed in both classes, appeared due to the in general low frequency pattern of MRI. Hence the first peak was excluded from the analysis.

### RUSBoost classification: 1p/19q co-deleted Vs. 1p/19q non-deleted glioma

We trained the dataset with different classification algorithms including classical machine learning models- for example, support vector machine (SVM), Naive Bayes, logistic regression, as well as ensemble models like LogitBoost and RUSBoost (Fig. 5). In our study the data imbalance problem was handled using RUSBoost classifier^9^ where the average training accuracy of 99% was obtained. The training accuracy obtained using traditional SVM, Naive Bayes, Logistic regression, RUSBoost and Logit-Boost is tabulated in Table 1. K-fold cross validation (*k=10*) was performed that yield 87% accuracy in classifying 1p/19q co-deleted and non-deleted G-III glioma. However the accuracy was reduced to 84% for G-II glioma cases. The performance measures like precision, recall, F-score and accuracy, obtained from RUSBoost classifier were reported in Table 2. It was found that the current approach improves the classification performance when it was compared with the result of our prior publication that utilizes frequency space texture measures to quantify texture pattern in 1p/19q co-deleted and non-deleted glioma subjects^7^.

**Table 1:** Training accuracy using different classifiers.

Classifiers	Training accuracy	
	G-II	G-III
Logistic regression	58%	66.67%
SVM	53%	66.52%
Naive Bayes	60%	62.87%
Logit boost	96.38%	91.52%
RUSboost	100%	98.5%

**Table 2:** Performance measures using RUSboost classifier on test data using LSPSD.

Grade	LGG Status	Precision	Recall	F-score	AUC
G-III (T1-W)	1p/19q co-deleted	0.85	0.9	0.89	87%
	1p/19q non-deleted	0.90	0.85	0.83	
G-III (T2-W)	1p/19q co-deleted	0.85	0.9	0.89	87%
	1p/19q non-deleted	0.90	0.85	0.83	
G-II (T1-W)	1p/19q co-deleted	0.88	0.79	0.89	84%
	1p/19q non-deleted	0.79	0.88	0.79	
G-II (T2-W)	1p/19q co-deleted	0.83	0.84	0.87	84%
	1p/19q non-deleted	0.84	0.83	0.78	

## Discussions

In this study, we proposed a novel methodology to determine the 1p/19q co-deletion status from T1C and T2-W S-MRI using cross correlation-periodogram model. Since it is evident that glioma, results due to mutation has better clinical outcome than those that occurred not due to mutation^10^, detecting the mutational status in glioma is extremely essential for the treatment prognosis. Although FISH test is the current gold standard for detecting chromosomal abnormalities, it suffers from some of its crucial limitations that prevent its application from regular use in diagnostics^11,12^. Alternatively, there are several imaging studies that have shown their potential to predict the same from conventional MRI and other advanced imaging modalities such as DWI, PWI, PET etc. For example in the paper of Iwadate et al^13^ the authors concluded that 11C–methionine PET might aid in discriminating tumors with and without 1p/19q co-deletion preoperatively. Brown et al.^14^ has shown textural measurements that can be helpful in discriminating tumors with and without mutation. Fellah et al.^5^ presented multi-parametric MRI to identify the mutational status, however the result showed no marked differences between tumors with and without 1p/19q co-deletion. Jansen et al.^6^ also derived several biomarkers using PET images to predict 1p/19q mutational status. But none of these biomarkers reliably could detect the same in individual study subjects.

Contrary to the studies reported in literature^3–6,12–14^, in this paper we have presented a different processing methodology that could assess the change in volumetric tumor heterogeneity by means of cross correlation-periodogram model in order to determine the presence of mutation non-invasively using S-MRI images. The consistency in VoCC pattern across slices clearly reveals the across-slice homogeneity for 1p/19q co-deleted glioma cases. On the other hand the randomness in VoCC pattern across slices reveals across-slice heterogeneity for 1p/19q non-deleted glioma cases. LSPSD estimate was computed on obtained VoCC to get suitable features for classification. The LSPSD estimate reveals static periodic pattern across volumetric glioma slices for majority of gliomas, occurred due to 1p/19q co-deletion. However it was observed that the periodic nature changes across slices in case of 1p/19q non-deleted glioma subjects. This is captured by extracting three different LSPSD features which are (i) difference energy between two periodograms, (ii) total volumetric energy and (iii) cut-off frequency of LSPSD. To overcome the limitations of imbalanced dataset RUSBoost classification was performed on extracted features. As seen from the results (Table 2), our proposed method was able to classify T1-W 1p/19q co-deleted and non-deleted glioma with 87% (G-III) and 84% (G-II) accuracy. The misclassification rate was higher in classifying the test data with 1p/19q co-deletion as compared to 1p/19q non-deletion. This may occur due to the error in FISH test. As reported in literature, the reliability of FISH test is 95% and 87.5% for the prediction of 1p and 19q deletions, respectively^2^. The second reason of misclassification might be due to the skewness of the dataset. In the dataset considered here, 64.44% of the instances belong to one class (1p/19q co-deletion) that lead to lower specificity when using RUSboost classifier as it gives more weights to minority class (1p/19q non-deletion) than majority class. However while comparing our approach with state-of-the-art studies it is seen that the current study outperforms the result reported by Z Akkus et. al^12^ that used same TCIA database and multiscale CNN approach. The classification accuracy obtained from their study was 75.6% (T2-W) and 63.3% (T1C) using no data augmentation. The obtained results outperformed another study reported in literature^5^ where the authors predicted the 1p/19q mutational status with 40% and 48% misclassification rates using multimodal MRI images and conventional MRI respectively. The result of our proposed methodology is comparable with the result reported by Tamim Niazi et al.^15^, where 82.43% classification accuracy was obtianed in determining 1p/19q co-deletion status. In this paper the authors used Radiomics features for the same TCIA LGG subjects. The result of the current study is also comparable with the result of our previously published work that utilized source distribution of VoCC to classify glioma sub-types^16^.

The availability of limited data size was one limitation of the current study. Also it was found that the heterogeneity increases with increase in glioma grades which might lead to some misclassifications between low-graded wildtype and high-graded mutant subjects. Hence we suggest future additional investigations such that the current findings can be well-validated for a large pool of patients.

## Conclusion

In this study, we present a new non-invasive method to estimate the 1p/19q chromosomal arm co-deletion status by quantifying textural characteristics using cross-correlation-periodogram model across selected MRI glioma slices. The proposed method provides promising results in classifying glioma with and without 1p/19q co-deletion. Robust classification of mutated and wildtype glioma based on the glioma texture, of course, require future validation, but these preliminary results point towards the promise of a future prognostic and predictive non-invasive MRI marker of glioma mutation for computer aided early diagnosis of brain tumor.

## Proposed Methodology

In our study the detection of 1p/19q co-deletion status is determined by the analyzing S-MRI images of considered glioma subjects as discussed in Sect. 2.1 and described in the subsequent subsections.

### Glioma segmentation and data normalization

The glioma portion was extracted from MRI using the ground truth provided in TCIA database. Here the ground truth images was utilized as a mask to segment the whole glioma. Post segmentation, each glioma image was normalized by z-scores using Eq. 1 in order to balance the intensity.

1 $$\begin{aligned} z=\frac{X(i,j)-\mu }{\sigma } \end{aligned}$$

where, $\mu$ and $\sigma$ denotes the mean and standard deviation of the image *X*(*i*, *j*) respectively.

### Detection of tumor tissue heterogeneity across slices by VoCC

The change in tumor heterogeneity across slices was investigated by means of cross correlation (CC) that evaluate whether two successive slices, (here, glioma ROI) of MRI image volume exhibit common features, and therefore are correlated. Hence CC analysis is likely to reveal if the differences in molecular characteristics of glioma lead to differences in structural layout across slices.

Given two successive glioma slices X and Y, the 2D cross correlation function is defined as

2 $$\begin{aligned} C(i,j) = \sum _{m=0}^{M-1} \sum _{n=0}^{N-1} X(m,n) Y(m-i,n-j) \end{aligned}$$

where, $$-(M-1) \le i \le (M-1)$$ and $$-(N-1) \le j \le (N-1)$$

In order to assess the change in tumor volume heterogeneity between two successive slices, we have proposed a new function *“Variance of CC” (VoCC)* that examines the change in CC for different values of lag. The VoCC was derived as:

3 $$\begin{aligned} \sigma _{CC}^{2}=\frac{\sum _{i=1}^{2M-1}(C(i,j)-{\overline{C}} (\bullet ,j))^{2}}{2M-2} \end{aligned}$$

where,

$$\begin{aligned} {\overline{C}}(\bullet ,j) = \frac{1}{2M-1} \sum _{i=1}^{2M-1} C(i,j) \end{aligned}$$

Since VoCC quantifies the change in uniformity of the intensity values across successive slices, this measure is relevant in examining the volumetric behaviour (across-slice behaviour) between mutant and wildtype glioma. One application of the proposed VoCC was reported in our previous publication^16^ where the source distribution of VoCC was computed in order to check if the significant differences exist between two glioma classes.

### Feature extraction

#### Examining the presence of 3D periodicity in 1p/19q co-deleted and non-deleted glioma

It was observed that the obtained VoCC for the two classes showed marked visible differences between the two glioma classes, with and without 1p/19q co-deletion. The essence of these visible differences were captured by extracting suitable features that would be useful for classification between mutant and wildtype glioma. As discussed before, in our prior work^16^ the source distribution of VoCC showed significant differences between two glioma sub-types. In the current study the applicability of VoCC to assess glioma heterogeneity is further investigated by determining its volumetric periodicity. In this papet the power spectral density (PSD) estimate of the VoCC corresponding the two classes was computed using Lomb-Scargle power spectral density (LSPSD) estimate in order to illustrate the differences in spectral signature of two classes. Lomb (1976) and Scargle (1982) postulated the Lomb-Scargle periodogram; an algorithm that helps in detection and characterization of periodicity^17,18^. There are few works reported in literature that utilized LS periodogram in order to find periodic patterns in the field of genetics and biological rhythmic process^19–22^.

The LS periodogram was formulated as below:

4 $$\begin{aligned} P_{LS}(f) = 0.5\frac{\sum _{n}\sigma _{CC}^{2}Cos(2\pi f [t_{n}-\tau ])^2}{\sum _{n}\sigma _{CC}^{2}Cos^{2}(2\pi f [t_{n}-\tau ])} + 0.5\frac{\sum _{n}\sigma _{CC}^{2}Sin(2\pi f [t_{n}-\tau ])^2}{\sum _{n}\sigma _{CC}^{2}Sin^{2}(2\pi f [t_{n}-\tau ])}) \end{aligned}$$

where, $\sigma _{CC}^{2}$ is VoCC which is a function of ’t’, given by Equation 3, $\tau$ is the time delay and is specified for each frequency ’f’ to ensure time shift invariance: $\tau = \frac{1}{4\pi f}tan^{-1}{\frac{\sum _{n}Sin(4\pi t_{n})}{\sum _{n}Cos(4\pi t_{n})}}$

Volumetric 3D periodicity of each LGG subject was measured by taking the inner product of periodogram of VoCC between two successive slices. If the VoCC plots of successive slices exhibit different pattern, The inner product of the respective periodogram will exhibit the following nature:

- The inner product of two corresponding spectrum will be significantly different compared to the input spectrum unequal no. of peaks.
- Corresponding peaks of two periodogram may not occur at similar location.
- There is large difference in corresponding peak amplitude of two spectrum.

As a result the corresponding peaks of two VoCC may not coincide at similar location. Also there will be large difference in corresponding peak amplitude of two periodogram. This will result a nearly flat spectrum with reduced oscillation.

Conversely, dominant peak will be visible in both the spectrum at same location when VoCC across slices exhibits similar pattern. There will be equal number of peaks and corresponding peaks of two periodogram will occur at similar location. The corresponding peak amplitude of two spectrum will also be nearly equal. As a result, the inner product of respective periodogram will also show a similar profile as the input spectrum with comparatively more oscillations.

The above concept of determining the change in 3D periodicity across MR slices was executed for each LGG subject to predict the presence of 1p/19q co-deletion. We hypothesize, *the change in periodic pattern across slices is negligible for cases with 1p/19q co-deletion*. The change in volumetric periodicity is quantified by extracting suitable spectral features for classification. The extracted spectral features include:

(i) Difference energy between two periodograms,
(ii) Total volumetric energy: It is defined as the total energy of the inner product of two peridograms.
(iii) Cut-off frequency of 3D LSPSD: It is defined as the frequency at which the amplitude of LSPSD estimate is nearly equal to zero.

### RUSBoost Classification

The dataset considered in our study is poorly balanced with a ratio of 2:1 (mutated:wildtype). In such cases constructing an effective classification model is a challenging task. When examples of a specific class greatly outnumber the examples of another class (data imbalance), the performance of traditional machine learning classification models drop significantly. These algorithms tend to only predict the majority class (negative class) data where the minority class (positive class) are treated as noise and are often ignored. Thus, there is a high probability of misclassification of the positive class by classifying all instances as negative class. Two most commonly used techniques in order to improve this class imbalance problem are data sampling and boosting^9^. The class distribution is balanced by sampling technique that either removes samples from the majority class (under-sampling) or add samples to the minority class (oversampling). Alternatively, boosting is an advance data sampling technique that can improve the performance of any weak classification model by iteratively building an ensemble models. In each iteration step, the weights of the sample which were incorrectly classified during the current iteration are modified. Such technique is very effective when dealing with class imbalance problem where the higher weights are given to the minority class examples which are likely to be misclassified in subsequent iterations. RUSBoost is one example of hybrid sampling/boosting algorithm that incorporates random undersampling (RUS)- a technique that removes data samples randomly from the majority class^9^.

Let ’n’ examples in dataset ’V’ are represented by tuple $(x_{k},y_{k})$ where $x_{k}$ is a point in feature space ’X’, and $y_{k}$ be the class label in a set of class label ’Y’. The algorithm begins with initializing the weight of each example to 1/n where ’n’ is the number of training examples. If the total number of iterations are denoted by ’P’ (represents the number of classifiers in the ensemble model), then P weak hypothesis $H_{t}$ are iteratively trained (t = 1 to P) using some classification algorithm ’*WeakLearn*’ as follows: First, RUS removes majority class examples until both the minority and majority class examples are balanced (1:1). This will result a new training dataset $V_{t}^{'}$ having a new weight distribution $W_{t}^{'}$. In the next step, $V_{t}^{'}$ and $W_{t}^{'}$ are passed to ’*WeakLearn*’ (base learner) in order to create the weak hypothesis $H_{t}$. Based on actual training dataset ’V’ and weight distribution ’$W_{t}$’, the pseudo-loss $\delta _{t}$ is calculated. After this, the distribution of weights for the next iteration $W_{t+1}$ is updated using weight update parameter $a_{t}$ followed by normalization. Finally, after ’P’ iterations the study hypothesis $\Theta (x)$ is returned as a weighted vote of the each weak hypothesis.

## Data Availability

The dataset analysed during the current study are available in The Cancer Imaging Archive (TCIA) repository. The dataset can be downloaded from this link: https://wiki.cancerimagingarchive.net/display/Public/LGG-1p19qDeletion#bef9e2ed4c354a92bae9ff35e8449e31
